# Symptom Cluster-Matching Antidepressant Treatment: A Case Series Pilot Study

**DOI:** 10.3390/ph14060526

**Published:** 2021-05-31

**Authors:** Sławomir Murawiec, Marek Krzystanek

**Affiliations:** 1 Harmony Medical Centre, Rondo ONZ 1, 00-124 Warszawa, Poland; smurawiec@gmail.com; 2 Clinic of Psychiatric Rehabilitation, Department of Psychiatry and Psychotherapy, Faculty of Medical Sciences in Katowice, Medical University of Silesia, Ziołowa 45/47, 40-635 Katowice, Poland

**Keywords:** symptom clusters, depression, personalized treatment, antidepressants, mechanism of action, serotonin, norepinephrine, dopamine, augmentation, treatment resistant depression

## Abstract

Despite treating depression with antidepressants, their effectiveness is often insufficient. Comparative effectiveness studies and meta-analyses show the effectiveness of antidepressants; however, they do not provide clear indications as to the choice of a specific antidepressant. The rational choice of antidepressants may be based on matching their mechanisms of action to the symptomatic profiles of depression, reflecting the heterogeneity of symptoms in different patients. The authors presented a series of cases of patients diagnosed with depression in whom at least one previous antidepressant treatment was shown to be ineffective before drug targeted symptom cluster-matching treatment (SCMT). The presented pilot study shows for the first time the effectiveness of SCMT in the different clusters of depressive symptoms. All the described patients obtained recovery from depressive symptoms after introducing drug-targeted SCMT. Once validated in clinical trials, SCMT might become an effective and rational method of selecting an antidepressant according to the individual profile of depressive symptoms, the mechanism of their formation, and the mechanism of drug action. Although the study results are preliminary, SCMT can be a way to personalize treatment, increasing the likelihood of improvement even in patients who meet criteria for treatment-resistant depression.

## 1. Introduction

Antidepressants, despite proven clinical efficacy [1] in a large proportion of patients, are not effective or their effectiveness is not much different from placebo [2,3,4,5]. Additionally, the problem of ineffectiveness in treating depression increases with age. The effectiveness of treating depression in the elderly population is two times lower than in people in early and middle adulthood, and there is essentially no difference between the efficacy of an antidepressant and a placebo [6]. Although meta-analyses provide clear evidence of effective antidepressants, they average the outcomes of individual patients and do not provide guidance on the choice of antidepressant. Such rational criteria for selecting a given antidepressant in an individual patient are needed by both psychiatrists and general practitioners in their clinical practice.

In a situation where the effectiveness of an antidepressant is insufficient, various management strategies are possible, such as changing the drug, adding a second antidepressant, or augmenting the effect of the antidepressant either with other drugs, or non-pharmacological biological treatments or psychotherapy [3,4,7,8]. However, augmentation methods are also of limited effectiveness. For example, electroconvulsive therapy, despite the improvement achieved during the electroshocks, has no lasting effect, and even despite continued pharmacotherapy, relapses are observed in a large (37%) proportion of patients; transcranial direct current stimulation and repetitive transcranial magnetic stimulation have only moderate and short-term efficacy in improving mood and cognitive functions in people with depression; deep brain was studied on small study groups, and light therapy has little effectiveness in enhancing the antidepressant effect of antidepressants in recurrent depression [9,10]. Therefore, in multi-module treatment, combining drugs, psychotherapy, and non-pharmacological biological forms of treatment [4] it is justified to search for new methods of augmentation of the effect of treating depression [11].

**Figure 1:**
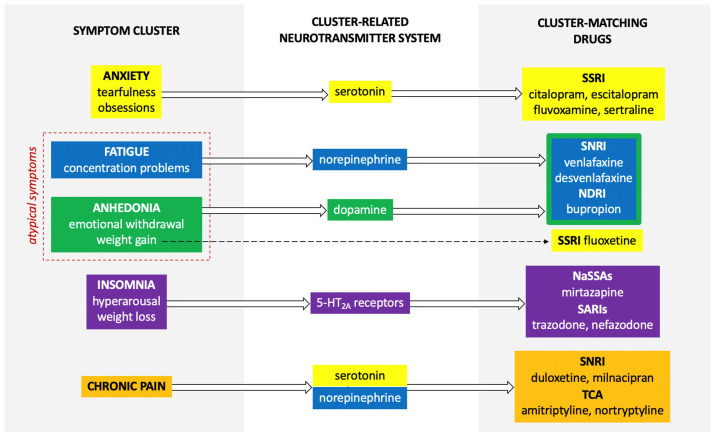
Relation between possible symptom origin and choice of drug. Anxiety symptoms are related to the serotonin deficiency syndrome, fatigue is associated with the norepinephrine deficiency syndrome, and anhedonia is associated with the dopamine deficiency syndrome. Dysregulation in the brain stem, related to 5-HT2A receptors is responsible for insomnia symptoms. Pain is mediated by both serotonin and norepinephrine, potentiating the effect of the endogenous opioid system. Drugs matching the system clusters are supposed to be more effective in the treatment of depression subtype with the predominance of the specific symptom cluster. SSRI—selective serotonin reuptake inhibitors; SNRI—serotonin norepinephrine reuptake inhibitors; NDRI—norepinephrine dopamine reuptake inhibitors; NaSSAs—noradrenergic specific serotonergic antidepressants; SARIs—serotonin antagonist reuptake inhibitors; TCA—tricyclic antidepressants.

A distinct and rarely used clinical method of augmenting antidepressant therapy is symptom clustering and matching the antidepressant to the patient’s symptomatic profile [12]. The identification of an individual profile of depressive symptoms and matching the mechanism of drug action to it may be a clear criterion for drug selection (Figure 1) [11]. Although the symptom cluster-based approach may be an effective method of increasing the effectiveness of antidepressant drug treatment, in practice, the evidence for the effectiveness of such an approach is limited [11,13].

A recent analysis of treatment efficacy predictors indicates that the optimization of treatment is to consider the clinical features of depression [14]. As the authors did not find in the medical literature clinical reports on SCMT in the treatment of patients who did not achieve sufficient improvement in the pharmacological treatment of depression, it was decided to describe a series of cases that may be the preliminary evidence of the effectiveness of this method of selecting drugs in depressive patients who had not previously improved. Demonstrating the effectiveness of this method in patients who have not achieved a satisfactory improvement in previous antidepressant treatment could contribute to the initiation of clinical trials of this method.

## 2. Results

**Figure 2:**
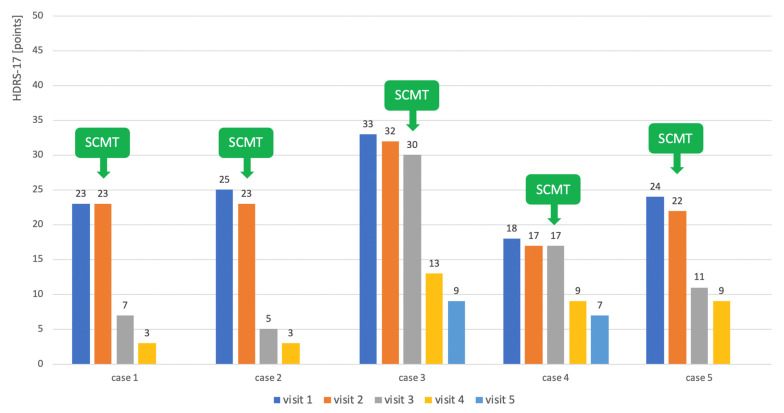
Changes in the intensity of depressive symptoms at subsequent visits before and after the introduction of symptom cluster-matching treatment (SCMT) with antidepressant. The severity of depression was measured with a 17-point Hamilton Depression Rating Scale (HDRS-17).

In each case of treatment after the ineffectiveness of the preceding antidepressant treatment, the use of symptom cluster-matching antidepressant treatment resulted in an improvement in depressive symptoms. The results are shown in Figure 2.

Individual case reports are summarized below.

### 2.1. Case 1—Anxiety/Irritability Cluster Depression

A 36-year-old man was treated for depression for the first time in his life. He was diagnosed with a severe depressive episode (HDRS-17 23 points). He was given lorazepam on an ad hoc basis due to anxiety and trazodone at the target dose of 150 mg. Lorazepam was prescribed for the acute relief of anxiety and was used by the patient for the first 2 weeks of treatment. At the dose of 100 mg in the evening, there was sedation in the morning, and the drug was discontinued. At the next visit, he continued to complain of mental tension, anxiety and irritability, insomnia, and obsessive thoughts. He also felt sadness and lack of energy and complained of a decline in activity. He said: “I am constantly sad, I have no strength for anything, I go to work, but I do it because I have to, I wake up around 2–3 a.m. and then I do not fall asleep until morning. But the worst is the irritability, anxiety, and tension. I get nervous with every event, then I experience for hours that I should react differently, every little thing throws me off balance, this is the worst.” Moreover, the patient complained about problems with concentration and impaired fresh memory. The severity of depression in HDRS-17 was 23 points. The patient received escitalopram at a target dose of 10 mg in the morning. At the next visit after 5 weeks, he reported a significant improvement in well-being (HDRS-17 7 points). He said: “I do not worry so much anymore, I do not get so nervous anymore, the same situations don’t make me so vibrate anymore, there is no fear that everything will collapse in a moment.” After another 6 weeks, the intensity of depression on the HDRS-17 scale was 3 points. During the described treatment period, the patient did not report any side effects associated with pharmacological treatment.

### 2.2. Case 2—Fatigue/Anhedonia Cluster Depression

A 42-year-old woman was previously not treated psychiatrically. She was diagnosed with an episode of severe depression (HDRS-17 25 points). The patient received moclobemide at the target dose of 300 mg/day. Despite the treatment, at the next visit, she reported that her mental state did not improve (HDRS-17 23 points). The patient said: “I am completely different than I was 2 months ago. Now I’m still sad, I would cry every moment, sometimes cry, I don’t know why, but I can’t show it in front of my baby. I have no strength for anything, I have no energy at all, as if someone sucked it out. And there was so much I could do during the day in the past. Breakfast and getting dressed for school takes three times as much time as before. I’m still afraid of something, now I don’t know what.” The patient received sertraline at the target dose of 100 mg in the morning. For the next visit after 6 weeks, the patient reported a significant improvement (HDRS-17 5 points). She said: “My mood is much better and I want to do something again. And it even gives me pleasure, just like it used to be. I am much calmer, I am no longer nervous about those things and troubles. I find it easier to sleep. At work, I can concentrate, I think more logically. Again, I am not yelling at my child, I have more patience, it is more difficult to upset me. This drug calmed me down pretty quickly, overall it’s better. It gives me satisfaction again everything I do.” At the next follow-up visit, the remission of depression was maintained (HDRS-17 3 points). During the described treatment period, the patient did not report any side effects associated with pharmacological treatment.

### 2.3. Case 3—Anhedonia/Fatigue Cluster Depression

A 46-year-old patient was diagnosed with a severe depressive episode (HDRS-17 33 points). He was given 10 mg escitalopram in the morning. After 5 weeks, there was no improvement (HDRS-17 32 points). Treatment was gradually changed to paroxetine at the target dose of 20 mg. At the next visit after 5 weeks, the patient talked about a slight improvement in anxiety but still complained of emotional indifference, significant depression, and lack of energy. He said, “I can’t do anything. The simplest thing takes me all day. I need several days to make one call. I don’t get dressed until afternoon. I don’t even have the strength to take care of myself. I only feel pain and despair. Before dawn, when I wake up, I’d rather it be over once and for all. I gave up everything that was of interest to me so far. Everything is indifferent to me, I am completely unresponsive to what is happening around me. I wake up at night and then a few hours earlier than before. In the morning, I feel terrible. I stopped eating and I lost weight. It occupies my thoughts all the time, everything else doesn’t matter.” The severity of depression in HDRS-17 was 30 points. The patient received venlafaxine at a target dose of 225 mg. After 6 weeks, there was a significant improvement in depressive symptoms (HDRS-17 13 points). He said, “It’s better. There is an improvement, I even start smiling sometimes. I feel better, all that has been is almost gone. I plan to go back to work.” At the next visit, the severity of depression in HDRS-17 was 9 points. During the described treatment period, the patient did not report any side effects associated with pharmacological treatment. 

### 2.4. Case 4—Insomnia Cluster Depression

A male, 63 years old, was treated for recurrent depression for 15 years. Another deterioration of his mental state took place in the fall of 2020. In addition to lowering the mood and apathy, he complained of anxiety, agitation, and restlessness, which prevented him from dealing with normal everyday activities. His appetite decreased with weight loss; besides, he had significant problems with falling asleep and woke up 2–3 h earlier than usual, unable to fall asleep. The severity of depressive symptoms as measured by the HDRS-17 scale was 18 points. The patient said, “I am at a critical moment—the fact that I cannot fall asleep, that I do not sleep through the night turns my entire next day into a nightmare. I don’t feel like doing anything, I can’t focus on anything. This life has no taste for me and I still want to enjoy life”. Then, the patient received escitalopram at a dose of 10 mg, but after 5 weeks, the intensity of depression did not change (HDRS-17 17 points). At that time, the drug was changed to venlafaxine at the target dose of 150 mg; however, after another 6 weeks, the mental state did not improve. Then, the treatment was changed to mirtazapine at a dose of 30 mg at night. His condition improved after approximately 5 weeks of treatment (interview data). At the visit 7 weeks after the initiation of mirtazapine, the severity of depression in HDRS-17 was 9 points. After another 6 weeks, the remission of depression was maintained (HDRS-17 7 points). The patient tolerated the treatment well.

### 2.5. Case 5—Chronic Pain Cluster Depression

A 50-year-old woman had been treated for depression for 12 years. At the first visit, when she reported relapse of depression, the intensity of symptoms in HDRS-17 was 24 points. She was given citalopram at the 40 mg target dose as she had previously taken it with good results. After 6 weeks, there was no improvement in depressive symptoms (HDRS-17 22 points). The patient said: “I take the best painkillers available, yet I feel pain every day. Despite the constant use of painkillers, the pain worsens, it is intrusive, because of it I wake up at night. It’s got to the point where I don’t sleep well every night. I fall asleep fearful of pain, and around 3 a.m., I wake up and feel only joint pain, dull, limiting, stiffening, from which there is no escape.” She also complained of sadness, anxiety, fatigue, and difficulty concentrating. She received duloxetine at a target dose of 60 mg in the morning. At the next visit after 4 weeks, the patient reported: “After a week, I finally slept through the night without pain. I got up without pain, got dressed without pain. I drove the car painlessly. I was able to reach for the things on the shelf, reach out, and take them off without pain. I had the strength to survive the day.” She stopped taking painkillers, her mood and drive improved, anxiety and concentration problems were gone. The severity of depression on the HDRS-17 scale was 11 points. At the 6-week follow-up visit, the improvement was maintained (HDRS-17 9 points). During the described treatment period, the patient did not report any side effects associated with pharmacological treatment.

## 3. Discussion

To the best of the authors’ knowledge, this case series is the first clinical report of SCMT in depressive patients who did not improve with a previous antidepressant treatment trial. As SCMT turned out to be effective for each patient, representing individual depressive clusters, reflecting the heterogeneity of clinical pictures of depression, our results may be the basis for a hypothesis that SCMT may be an effective method of personalized treatment in psychiatry. To verify this clinical hypothesis, the results of this pilot study must be confirmed in subsequent clinical trials. These studies should be placebo-controlled and carried out on larger groups of patients, representing each of the depressive clusters. Although the presented results are only preliminary, they seem to justify the need for such research.

The heterogeneity of depressive symptoms and thus the possible variety of depression subtypes make it difficult to test the effectiveness of its treatment with antidepressants [12]. Different types of depression probably require different treatments, and their effectiveness may vary depending on the severity of the depressive symptoms. The choice of an antidepressant itself is influenced by many factors, such as the patient’s clinical symptom profile, the physician’s knowledge and experience, preferences, treatment styles related to time and place, CPGs that change with the progress of clinical trials and their understanding by the physician, and even doctors’ relations with pharmaceutical companies [12]. In clinical practice, there are no clear guidelines for choosing a given drug in a given patient. 

This approach may be facilitated by the symptom cluster-matching antidepressant treatment. The described cases may show that this method may be effective in the case of not sufficient effect of antidepressant treatment, even in the case when the patient meets the criteria for resistant depression (case 3 and 4). Importantly, symptom cluster-matching treatment may also be effective in elderly patients (case 4). We are fully aware of the limitations associated with presenting a case series study, but the results obtained by us should encourage clinical trials in which the choice of a depressive drug would be tailored to the specificity of depressive symptoms in individual patients.

Depression has individual characteristics in every patient, especially the number and severity of symptoms, and its treatment may need to be personalized [12]. The current approach in comparative effectiveness studies of antidepressants uses the criteria for diagnosing mental disorders included in classifications such as ICD and DSM, which unify and average the heterogeneity of depressive symptoms to determine the presence of a depressive episode. This may explain why the recommendations resulting from the conclusions of clinical trials and their meta-analyses are effective only in some patients and not in the majority, as might be expected. The lack of 100% clinical effectiveness of the approach unifying the heterogeneity of depression seems to undermine the validity of this method and prompts the search for a different drug selection strategy.

Moreover, some symptomatic areas commonly found in depression are ignored in the classifications, as exemplified by the absence of irritability, anger, and aggression dimensions in the criteria for the depressive syndrome. The irritability cluster of symptoms is frequently reported by patients in daily clinical practice [15] and was present in the description of case 1. This patient’s report was dominated by complaints of irritability and reduced stress tolerance. A review by Young et al. indicates that anger, irritability, and irritation may respond favorably to the administration of drugs with a strong serotonin effect [15]. This may indicate the need to distinguish, in addition to the five depression symptom clusters in the model described by Lin et al., the irritability cluster of depressive symptoms.

Another problem may be the inability of clinicians to distinguish individual features and dimensions of the depressive syndrome. Due to the commonly accepted, unifying nature of psychiatric classifications, clinicians may tend to adopt an analogous unifying approach in the process of diagnosing a depressive syndrome, which may flatten the profile of depressive symptoms and lead to diagnostic simplification in the form of diagnosing depression without analyzing the full range of symptoms and their individual context. Approaches based on the symptom cluster model can be a step in the right direction—it requires the clinician to listen carefully to the patient, in-depth analysis of patient-reported symptoms, and personalized psychiatry. 

The heterogeneity of depressive symptoms may be related to different mechanisms of their formation related to various monoaminergic neurotransmitters in the brain [16,17]. This may justify the need to define individual symptom clusters of patients in order to combine them with drugs with corresponding mechanisms of action through specific neurotransmission disorders. So far, such a pharmacodynamic thinking model linking the mechanism of drug action with symptomatic clusters of depression is not commonly used clinically [12]. The describes cases may show that this approach is successful in treating individual patients. Such a solution could be beneficial in a situation where the patient does not meet the criteria of any of the depression subtypes or when the severity of the symptoms of depression is disproportionate to the number and type of the symptoms. The introduction of this approach to drug efficacy analysis in clinical trials could prove its effectiveness and lead to significant progress in effectiveness depression treatment on an outpatient basis.

The symptom cluster-based approach in the treatment of depression appears to be consistent with daily clinical practice. As already shown in 2004 by Zimmerman et al., psychiatrists pay special attention to the presence of symptoms in depression such as anxiety, sleep disturbances, fatigue, anger or irritability, increased or decreased appetite, and melancholic or atypical symptoms [13]. Such an intuitive symptom cluster-based approach of practitioners may be indirect evidence that there is no one depression and one most effective antidepressant and that each case of depression should be treated differently. The studies of the frequency of depression symptoms indicate that anxiety, fatigue, insomnia, and pain are the most common, so they can be used as a basis for the symptom cluster treatment model, combining these symptoms with specific disturbances in a given monoamine neurotransmitter pathways, and then, depending on it, choosing drugs with the matching mechanism of action [11]. The use of this approach in the patient cases described by us turned out to be effective, which might indicate its effectiveness. However, as already mentioned, this requires confirmation in controlled clinical trials.

There is evidence of the effectiveness of symptom cluster-matching antidepressant treatment described in the literature. A similar approach to treating depression by identifying a symptom cluster in a patient was investigated by Chekroud et al. [18]. The study was an analysis of the results of previous clinical trials with the grouping of patients treated for depression according to their symptomatic clusters and reassessment of the effectiveness of the treatment. They used the model of symptom cluster approach, which was based on the Quick Inventory of Depressive Symptomatology scale and 14 items from the clinician-rated Hamilton Depression rating scale extracting three symptom clusters: insomnia, core emotional symptoms, and atypical symptoms [18]. Their analysis confirmed the validity of the symptom cluster approach. In their conclusion, antidepressants are more effective when analyzed in symptom clusters than when their effects are assessed globally in all patients [18].

In addition to analyzing the symptom of clusters at the time of initiation of treatment, a useful approach applied by us may be to predict the effects of a selected drug on symptom clusters and verify the effectiveness of treatment by assessing the presence of symptoms of this cluster at the next visit. Thus, symptom cluster-matching treatment should be based not only on the analysis of the symptom clusters mentioned above but should also include the next treatment time point. The assessment of the expected results should include the prediction of the results of the interaction between the symptoms revealed by a particular patient and the action profile of a particular drug, and on this basis, its effectiveness should be verified [19].

As already mentioned, the results presented by us should be critically assessed. The study was a case study series, was uncontrolled, and the number of cases is small. Due to the lack of a reference group, it is impossible to objectively exclude the placebo effect. However, in our opinion, the obtained results suggest that the use of the described cluster symptom matching treatment can be an effective way to increase the effectiveness of pharmacological treatment of depression, including treatment-resistant depression and depression in old age.

## 4. Methods

The criteria for selecting patients for the case series study were the diagnosis of a depressive episode according to the ICD-10 criteria, the predominance of depressive symptoms belonging to one of the following symptom clusters: anxiety/irritability, fatigue/anhedonia, insomnia, and chronic pain (according to Lin et al.) [11], and no improvement after antidepressant treatment, followed by drug-targeted symptom cluster-matching treatment. The exclusion criteria were taking psychoactive substances and meeting the criteria of the organic mental syndrome according to ICD-10.

Depression severity was assessed using a 17-point Hamilton Depression Rating Scale (HDRS-17). Assessment of the severity of depression was performed at intervals of 5 weeks +/− 1 week. For the case series study, two cases were selected for the cluster: fatigue/anhedonia, depending on the dominance of fatigue (fatigue/anhedonia) or anhedonia (anhedonia/fatigue) to represent both situations, occurring in depression. All patients were treated on an outpatient basis.

The clinical evaluation of the patients was always performed with all due diligence, and each patient was rated over the same amount of time. Symptom improvement was conceptualized according to the remission criterion defined for HDRS-17. During the treatment period described in the case reports, patients were not receiving any medications other than those described in the manuscript.

## 5. Conclusions

Once validated in clinical trials, cluster-matching antidepressant treatment may prove to be an effective treatment option in patients with depression who do not achieve clinical improvement or are resistant to treatment. The effectiveness of symptom cluster-matching antidepressant treatment presented in the described cases indicates so far only the possibility of a rational selection of an antidepressant, matching the symptom cluster with the drug’s mechanism of action. Future symptom-based drug-targeting studies can lead to clear practical criteria for personalized antidepressant choice.

## 6. Patents

This retrospective case series study used data from the treatment of three men and two women treated as outpatients by the authors for a depressive episode. Patient data were anonymized for this study, making it impossible to identify patients. All patients received treatment according to routine clinical practice for pharmacological treatment of depression. After reviewing the patient’s documentation, the authors selected five cases of depression in which the patients presented symptoms typical of symptomatic clusters of depression to present and discuss them as an example of using the symptom cluster-matching treatment method that both authors use on a daily basis.

## Data Availability

Data supporting reported results on the treatment of patients can be found in the authors’ medical records and can be obtained on demand from corresponding author.
